# Inhibition of HSP90 Promotes Neural Stem Cell Survival from Oxidative Stress through Attenuating NF-*κ*B/p65 Activation

**DOI:** 10.1155/2016/3507290

**Published:** 2016-10-12

**Authors:** Qian Liu, Yun Li, Wenkai Jiang, Yunzi Li, Lin Zhou, Bing Song, Xinfeng Liu

**Affiliations:** ^1^Department of Neurology, Jinling Hospital, Medical School of Nanjing University, 305 East Zhongshan Road, Nanjing, Jiangsu 210002, China; ^2^School of Dentistry, Cardiff Institute of Tissue Engineering and Repair, Cardiff University, Heath Park, Cardiff CF14 4XY, UK; ^3^State Key Laboratory of Military Stomatology & National Clinical Research Centre for Oral Diseases & Shaanxi Key Laboratory of Oral Diseases, Department of Operative Dentistry & Endodontics, School of Stomatology, Fourth Military Medical University, No. 145 Western Changle Road, Xi'an, Shaanxi 710032, China; ^4^Department of Chemistry & Chemical Biology, MSC03 2060, University of New Mexico, Clark Hall B58, Albuquerque, NM 87131-0001, USA; ^5^College of Chemistry and Materials Science, Jiangsu Key Laboratory of Biofunctional Materials, Nanjing Normal University, Nanjing 210023, China

## Abstract

Stem cell survival after transplantation determines the efficiency of stem cell treatment, which develops as a novel potential therapy for several central nervous system (CNS) diseases in recent decades. The engrafted stem cells face the damage of oxidative stress, inflammation, and immune response at the lesion point in host. Among the damaging pathologies, oxidative stress directs stem cells to apoptosis and even death through several signalling pathways and DNA damage. However, the in-detail mechanism of stem cell survival from oxidative stress has not been revealed clearly. Here, in this study, we used hydrogen peroxide (H_2_O_2_) to induce the oxidative damage on neural stem cells (NSCs). The damage was in consequence demonstrated involving the activation of heat shock protein 90 (HSP90) and NF-*κ*B/p65 signalling pathways. Further application of the pharmacological inhibitors, respectively, targeting at each signalling indicated an upper-stream role of HSP90 upon NF-*κ*B/p65 on NSCs survival. Preinhibition of HSP90 with the specific inhibitor displayed a significant protection on NSCs against oxidative stress. In conclusion, inhibition of HSP90 would attenuate NF-*κ*B/p65 activation by oxidative induction and promote NSCs survival from oxidative damage. The HSP90/NF-*κ*B mechanism provides a new evidence on rescuing NSCs from oxidative stress and also promotes the stem cell application on CNS pathologies.

## 1. Introduction

Stem cell transplantation is considered as a novel potential therapy for several central nervous system (CNS) diseases through both growth/trophic factor support and neural cell replacement [1–3]. However, the dramatic number of cell death after transplantation due to oxidative stress, inflammation, and immune response in lesion limits the therapy for wide application [4–7]. To increase the stem cell survival after transplantation, strategies against the above damaging pathologies are investigated in recent decades [8].

Among the damaging pathologies, oxidative stress is considered as a primary vital damaging factor to engrafted stem cells. The pathology directs the stem cells to produce extra reactive oxygen species (ROS), which could trigger several of intracellular signalling cascades, thus leading to DNA damage and cell apoptosis and even death [9]. Therefore, study on the underlying signalling mechanism in oxidative stress could provide some evidence to rescue the engrafted stem cells from damage. Previous study of ours reported the mediating role of NF-*κ*B/p65 in neuronal protection against oxidative stress [10] while both whether this signalling transduction also applies on NSCs survival and the pathway from oxidative stimulation to NF-*κ*B/p65 activation in NSCs have not been investigated before.

In this study, we investigated the role of heat shock protein 90 (HSP90) and NF-*κ*B/p65 in neural stem cells (NSCs) under induced oxidative injury, as well as their relationship during the pathology. According to our results, oxidative stress firstly triggers HSP90 in stimulated NSCs. The triggered HSP90 in consequence activates NF-*κ*B/p65 through IKK/I*κ*B/p65 cascade, which leads finally to cell death. Inhibiting HSP90 activity will block the downstream activation of NF-*κ*B/p65 under oxidative stress and increase NSCs survival. The HSP90/NF-*κ*B mechanism might rescue NSCs from oxidative stress and also promotes the stem cell study and application on CNS pathology.

## 2. Material and Methods

### 2.1. Reagents

DMEM/F12, B27 supplement, EGF, bFGF, Penicillin/Streptomycin, Accutase, Poly-D-lysine (PDL), and Annexin V-FITC/PI kit were purchased from Thermo Fisher Scientific (San Jose, CA, USA). Hydrogen Peroxide (H_2_O_2_), DMSO, Geldanamycin (GA), JSH-23, and MTT were purchased from Sigma-Aldrich (St. Louis, MO, USA). All primary antibodies used in this study were purchased from Cell Signalling Technology (Danvers, MA, USA). Goat anti-rabbit/mouse IgG (H+L) Alexa Fluor® 488/594 and HRP conjugate secondary antibodies were purchased from Thermo Fisher Scientific (San Jose, CA, USA). Hoechst 33342 and DCFH-DA probe were purchased from Beyotime Biotech (Haimen, China).

### 2.2. Neural Stem Cell Culture

All experiments were carried out in accordance with Animals (Scientific Procedures) Act 1986 under project license 30/2816 issued by UK Home Office. This study was approved by Jinling Hospital Research Ethics Committee. C57BL/6 mice were housed in a temperature-controlled environment (22 ± 0.5°C) with a 12-h  light-dark cycle and allowed free access to food and water. All efforts were made to minimize animal suffering and reduce the number of animals used. The NSCs were dissected from embryonic brain tissue of E14 day C57BL/6 mice (mice were provided by Model Animal Research Centre of Jingling Hospital Nanjing, Jiangsu, China). Briefly as described previously [11], the brain tissue was transferred into ice bathed DMEM/F12 medium for meninx and vessel tissue removal. The brain tissue was then digested by Accutase. The single-cell suspension was collected and resuspended in DMEM/F12 medium containing B27, bFGF, and EGF. The cell culture was kept in at 37°C, 5% CO_2_. Changing culturing medium was done every three days. Passage of the NSCs was performed as the neurospheres grew to 50–100 *μ*m diameter.

### 2.3. NSCs Oxidative Stress Induction

The neurosphere culture was digested into single-cell suspension and seeded on the PDL precoated cover glass, dish, or 6-well plates, depending on further test. The NSCs were cultured at 37°C, 5% CO_2_ overnight. As far as the single cell of NSCs attached, change the medium with DMEM/F12 medium without B27, EGF, and bFGF for cell starving, preparing the following oxidative stress induction.

Following 2 h starving, 50, 100 (final applied moderate concentration for modelling), 200, and 400 *μ*M H_2_O_2_ were, respectively, added to DMEM/F12, culturing for further 2 h as the oxidative stress induction, to choose the final modelling dosage. DMEM/F12 medium without any supplements was used as the vehicle control.

### 2.4. NSCs Treatment

For the signalling inhibitor pretreatment, after the cell starving, 0.5 *μ*M (final concentration) GA and 8 *μ*M (final concentration) JSH-23 were administrated, respectively, to the monolayer NSCs for 1 h, before the oxidative stress induction. DMSO was used as the cosolvent, with the final concentration of 0.1%. DMEM/F12 medium containing 0.1% DMSO was used as the vehicle control.

### 2.5. Cellular Detection

The cell viability was assessed with MTT assay as previously described [10]. The intracellular reactive oxygen production was detected with the DCFH-DA probe dye, according to the manuscript's instructions. The apoptosis and cell death assessment were detected with Annexin V-FITC/PI kit following the manuscript's instructions.

### 2.6. Immunofluorescence (IF)

The NSCs were fixed with 4% paraformaldehyde (Sigma-Aldrich, MO, USA) and permeabilized with 0.1% Triton X-100 (Sigma-Aldrich, MO, USA). After blocking the nonspecific proteins with 3% BSA-PBS solution, the fixed cells were then incubated with anti-HSP90 and anti-phospho-p65 over night at 4°C. The cells were transferred to FITC/TRITC-conjugated secondary antibodies (Cell Signalling Technology, MA, USA), incubating for 1 hour. Fluorescence data was collected with fluorescence microscope and analyzed with ImageJ software.

### 2.7. Western Blotting

The cell lysate was collected from NSCs. The sample loadings were subjected onto 12% SDS-PAGE gel for electrophoresis as previously described with slight modification [10]. The protein bands were transblotted onto PVDF membrane (Millipore, OH, USA), being blocked in 5% BSA-TBST buffer and incubated with the primary antibodies and HRP-conjugated secondary antibodies. The antigen-antibody complexes were then detected with an ECL reagent kit (Millipore, OH, USA), and developed with X-ray film. The protein band density was analyzed with ImageJ software.

### 2.8. Statistical Analysis

We evaluated the statistical significance using independent-samples* t*-tests, followed by ANOVA tests when the data involved three or more groups. Results are presented as means ± SD.* P* < 0.05 was considered to be significant.

## 3. Results

### 3.1. Culture of Neural Stem Cells

**Figure 1:**
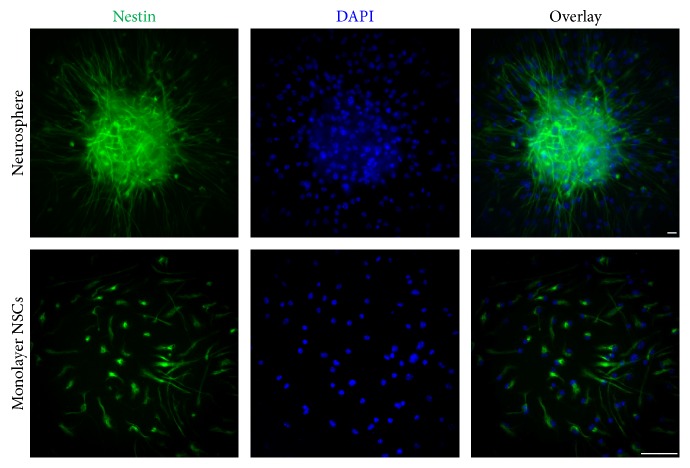
Identification of NSCs. Immunofluorescence identification of neurosphere and monolayer culture of NSCs with anti-Nestin. Scale bar: 20 *μ*m.

The NSCs were dissected from embryonic mouse at E14 and cultured to form the neurospheres in flasks. For cell identification, immunofluorescence targeting at the Nestin was applied (Figure 1). As the result demonstrated, up to ~95% cells were detected as Nestin-positive NSCs.

### 3.2. Oxidative Stress Induced Cell Death in Neural Stem Cells

The monolayer culture of NSCs was subjected to 100 *μ*M H_2_O_2_ treatment for 2 h. After oxidative stress induction, the cell morphology, MTT assay, DCF-DA, and Annexin V-FITC/PI staining were applied to assess the cell injury.

**Figure 2:**
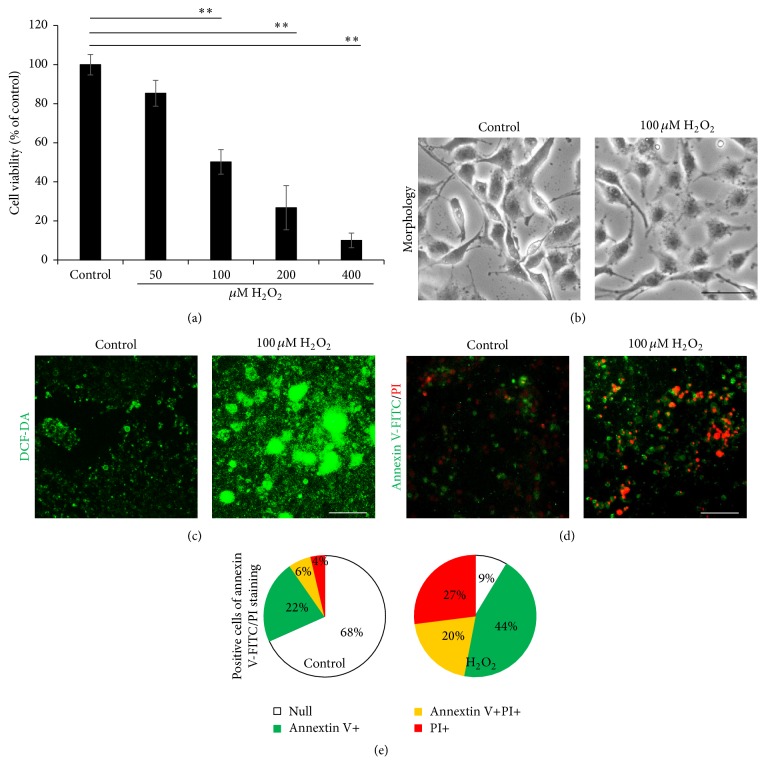
NSCs survival from oxidative stress induced by H_2_O_2_ treatment. The NSCs were subjected to 100 *μ*M H_2_O_2_ induction for 2 h, following cell starving within the DMEM/F12 medium without B27, EGF, and bFGF. The NSCs treated with the vehicle medium were used as control. (a) The cell viability by MTT assay of the NSCs. (b) The cell morphology of the NSCs. (c) The ROS production of the NSCs probed by DCF-DA. (d) The apoptosis and cell death of the NSCs detected by Annexin V-FITC/PI kit. (e) Quantified positive cell number of the NSCs with Annexin V-FITC/PI staining. ^*∗∗*^ *P* < 0.01 was considered to be significantly different between control and H_2_O_2_ groups.

According to the results with 50, 100, 200, and 400 *μ*M H_2_O_2_ treatment (Figure 2(a)), the injury with 100 *μ*M H_2_O_2_ was the moderate dosage to NSCs (cell viability proportion dropped to 50.2 ± 6.3%). While 50 *μ*M H_2_O_2_ induced a very light injury, 200 and 400 induced too heavy damage of NSCs to death, which are not the proper dosages for model establishment in this study. Therefore, dosage of 100 *μ*M was applied in the following experiments. With the morphology result (Figure 2(b)), the control NSCs displayed healthy cell morphology with bright and smooth cell body and extended neural synapses, while the injured NSCs by 100 *μ*M H_2_O_2_ treatment demonstrated broken cytomembrane, wizened cell body, and synapses, indicating an unhealthy status. DCF-DA staining demonstrated obviously more ROS production in H_2_O_2_ treatments NSCs, comparing to that in control NSCs (Figure 2(c)). While for Annexin V-FITC/PI assay (Figure 2(d)), 44% of the H_2_O_2_ injured NSCs were positively stained by Annexin V-FITC indicating an early stage apoptosis, which was significantly higher than that with control NSCs at a normal level of 22%; 20% of the injured NSCs demonstrated double staining by Annexin V-FITC and PI staining, also notably greater than that with the control NSCs at 6%; 27% of the injured NSCs were stained by PI indicating later stage of cell death, while the proportion in control NSCs was only 4%. The assay demonstrated a 9% healthy cell proportion in the H_2_O_2_ treatments NSCs, comparing with which was significantly higher in control NSCs, up to 68% (Figure 2(e)).

Taken together, these results suggested a significant damage on* in vitro* cultured NSCs by 100 *μ*M H_2_O_2_ treatment. The injury produced could be due to the oxidative stress induced apoptosis and cell death.

### 3.3. Oxidative Stress Triggered the Activation of HSP90 and NF-*κ*B/p65

To address the underlying molecular mechanism of cell survival of NSCs from oxidative stress induced apoptosis and cell death, the role of HSP90 and NF-*κ*B/p65 in the pathology was investigated.

**Figure 3:**
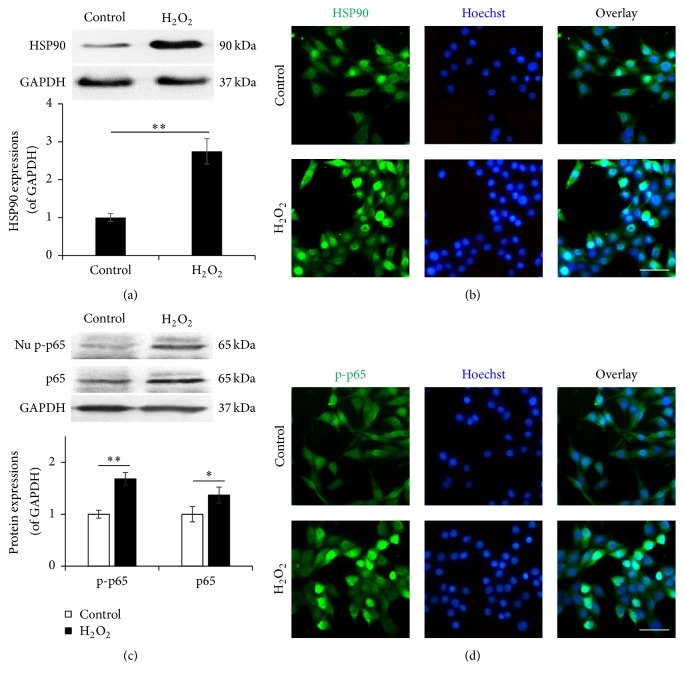
H_2_O_2_ triggers HSP90 and NF-*κ*B/p65 activation. (a) and (c) The protein expression of HSP90, nuclear phospho-p65, and total p65 in the NSCs induced by H_2_O_2_ induction. (b) and (d) The intracellular distribution of HSP90 and the nuclear translocation of phospho-p65 in the H_2_O_2_ induced NSCs. The protein expression and intracellular distribution in NSCs cultured within the vehicle medium were used as control. ^*∗*^ *P* < 0.05 and ^*∗∗*^ *P* < 0.01 were considered to be significantly different between control and H_2_O_2_ groups.* n* = 3.

With oxidative stress injury by H_2_O_2_ administration, the protein expression of HSP90 significantly boosted up to 1.7-fold higher than that in control NSCs (Figure 3(a)). Consistently, as immunofluorescence demonstrated, HSP90 displayed a mainly cytoplasmic distribution in control NSCs. The nucleus around expression was observed only within a small number of cells while a significant centralized expression of HSP90 around the nuclei was detected in greater number of the NSCs treated by H_2_O_2_ (Figure 3(b)). The results indicated an involvement of HSP90 in NSCs survival against oxidative stress injury.

As for NF-*κ*B/p65, the H_2_O_2_ administration induced significantly upregulation on both total p65 and nuclear phospho-p65, while the protein in control NSCs maintained on regular level (Figure 3(c)). The immunofluorescence also demonstrated an increased nuclear distribution of phospho-p65 after H_2_O_2_ administration, comparing with control NSCs (Figure 3(d)). These data suggest a notable activation of NF-*κ*B/p65 in NSCs survival against oxidative stress injury.

### 3.4. Geldanamycin Blocked Oxidative Stress Triggered NF-*κ*B/p65 Activation

Since both HSP90 and NF-*κ*B/p65 were involved in oxidative stressed injured NSCs, to determine their streaming relationship, pharmacological inhibitors targeting at the two signalling proteins were applied.

**Figure 4:**
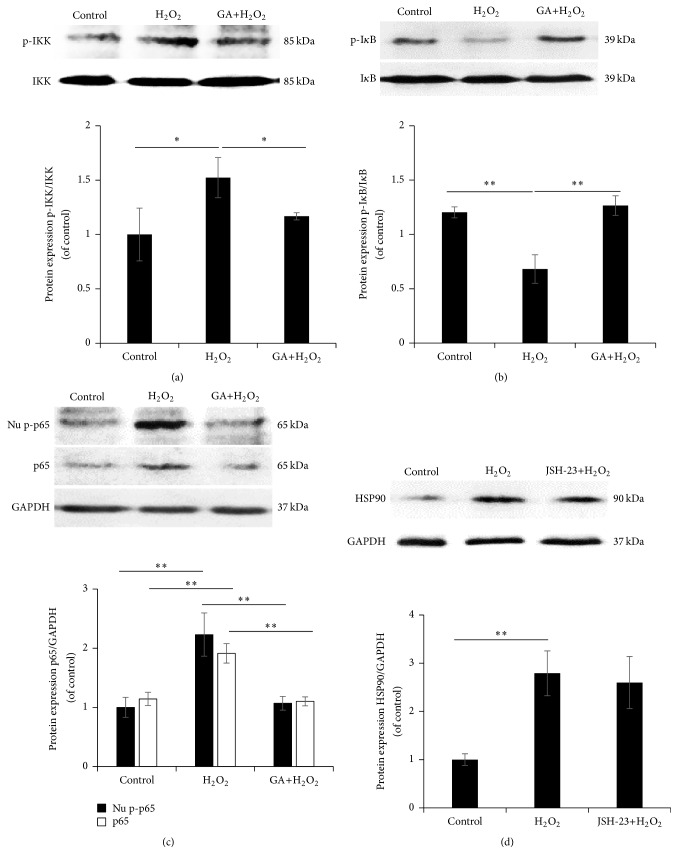
Effect of Geldanamycin and JSH-23 on H_2_O_2_ induced NF-*κ*B/p65 and HSP90 activation. The pretreatment of 0.5 *μ*M GA or 8 *μ*M JSH-23 was administrated to NSCs for 1 h, followed by the H_2_O_2_ induction. (a)–(c) The protein expression of phospho-IKK, total IKK, phospho-I*κ*B, total I*κ*B, nuclear phospho-p65, and total p65 in the NSCs treated with GA and H_2_O_2_. (d) The protein expression of HSP90 in the NSCs treated with JSH-23 and H_2_O_2_. The protein expression in NSCs cultured within the vehicle medium was used as control. ^*∗*^ *P* < 0.05 and ^*∗∗*^ *P* < 0.01 were considered to be significantly different between control and H_2_O_2_ or between H_2_O_2_ and GA/JSH-23+H_2_O_2_ groups.* n* = 3.

As the specific inhibitor of HSP90, the pretreatment of 0.5 *μ*M GA significantly abolished the H_2_O_2_-induced upregulation on phospho-IKK, phospho-I*κ*B, and nuclear phosphor-p65, which suggested a deactivating effect on NF-*κ*B/p65 activation (Figures 4(a)–4(c)).

### 3.5. JSH-23 Failed in Blocking the Oxidative Stress Triggered HSP90 Activation

While with the pretreatment 8 *μ*M JSH-23, the inhibitor of NF-*κ*B/p65, the H_2_O_2_-induced upregulation on HSP90 in NSCs maintained with the augmented expression level [12], indicating a failed regulation of the NF-*κ*B/p65 inhibitor on triggered HSP90 in oxidative stress injured NSCs (Figure 4(d)).

### 3.6. Induced Colocalization of HSP90 and NF-*κ*B/p65 in Oxidative Stress Injured Neural Stem Cells

According to the results from both inhibitors, HSP90 could potentially work as an upper-stream signalling over NF-*κ*B/p65 in the survival of NSCs from H_2_O_2_-induced neural oxidative stress.

**Figure 5:**
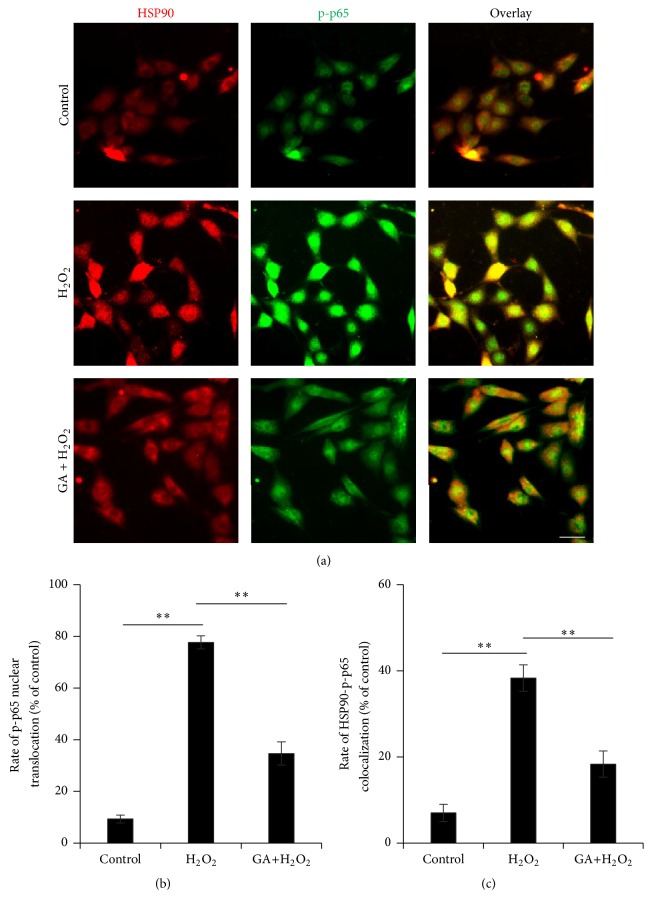
Effect of Geldanamycin on the colocalization in the H_2_O_2_ induced NSCs. (a) The protein distribution and colocalization of HSP90 and NF-*κ*B/p65 in control, H_2_O_2_ induced, and GA+H_2_O_2_ induced NSCs. (b) Percentage of phospho-p65 nuclear translocation in control, H_2_O_2_ induced, and GA+H_2_O_2_ induced NSCs. (c) Percentage of colocalization of HSP90 and NF-*κ*B/p65 in control, H_2_O_2_ induced, and GA+H_2_O_2_ induced NSCs. ^*∗∗*^ *P* < 0.01 was considered to be significantly different between control and H_2_O_2_ groups or between H_2_O_2_ and DA+H_2_O_2_ groups.* n* = 3.

Further investigation with GA demonstrated that, in contrast with the cytoplasmic distribution of HSP90 and nuclear expression of p-p65 at a low level in control NSCs, the H_2_O_2_-injured NSCs displayed a dramatic upregulation on p-p65 nuclear translocation, as well as a significant augment on HSP90 and p-p65 colocalization at nuclei (Figure 5). This diploid activation on HSP90 and nuclear p-p65 under H_2_O_2_ induction in NSCs was obviously abolished by pretreatment of GA, the inhibitor of HSP90. The protein distribution of HSP90, especially around the nuclei, was attenuated, while the nuclear translocation of p-p65 was notably decreased. Therefore, the colocalization of HSP90 and p-p65 was partly abolished by GA pretreatment (Figure 5).

The results indicated that as far as HSP90 activity was inhibited by GA, NF-*κ*B/p65 signalling was blocked as an effector. HSP90 activity performed as an upper-stream signalling of NF-*κ*B/p65 under H_2_O_2_ induction.

### 3.7. Protective Effect of Geldanamycin on Oxidative Stress Injured Neural Stem Cells

With the determination of upper-stream role of HSP90 over NF-*κ*B/p65, we further mean to confirm the function of HSP90 in NSCs survival from oxidative stress induced cell death.

**Figure 6:**
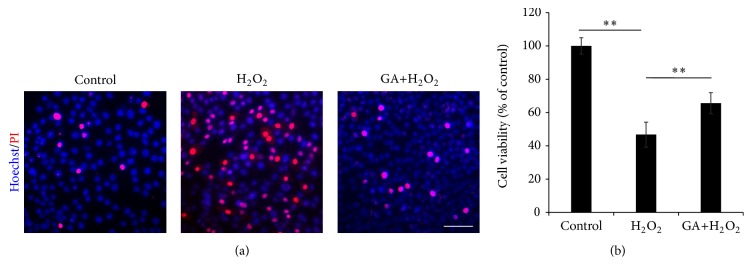
Effect of Geldanamycin on the H_2_O_2_ damaged NSCs. The pretreatment of 0.5 *μ*M GA was administrated to NSCs for 1 h, followed by the H_2_O_2_ induction. The NSCs treated with the vehicle medium were used as control. (a) The cell death of each group of NSCs detected by Hoechst 33342/PI double staining. (b) The cell viability of each group of NSCs by MTT assay. ^*∗∗*^ *P* < 0.01 was considered to be significantly different between control and H_2_O_2_ or between H_2_O_2_ and DA+H_2_O_2_ groups.* n* = 3.

Both Hoechst/PI double staining and MTT assay demonstrated the neuroprotective effect of GA pretreatment against H_2_O_2_-injury. With the Hoechst straining as a cell count, the PI staining positive cells were considered as the damaged NSCs, whose number was obviously decreased with GA treatment, followed by H_2_O_2_-injury; the cell viability was rescued from 46.7 ± 7.5% to 65.6 ± 6.3% with GA treatment, in previous of H_2_O_2_-injury (Figure 6).

The results suggested a neuroprotection of inhibiting HSP90 with GA on NSCs survival from oxidative stress.

## 4. Discussion

As the stem cell transplantation arising as a potential therapy for serials of CNS disease [2], the cell survival of engrafted stem cells after transplantation has become a vital limitation to the therapy outcome and further application [4]. The engrafted stem cells always face very complicated pathological condition, such as oxidative stress, inflammation, and immune response, among which oxidative stress could play a primary role [9, 13]. A range of CNS pathologies, such as neurodegenerative diseases and neural trauma, are symbolized as oxidative stress, following the overbalanced ROS production [6, 9]. The oxidative stress consequently induces the cell damage around the lesion area, as well as the engrafted stem cell. To overcome the neural oxidative stress and rescue the engrafted stem cell, investigation on the mechanism underlying stem cell survival from oxidative stress is quite needed. Our previous study has revealed that H_2_O_2_ could damage the neuronal cell line and PC12 cells at concentration of 400 *μ*M [10]; the ischemia/reperfusion injury, of which oxidative stress plays a crucial role, causes different types of cell death on neurons, including apoptosis and autophagy [8]. However, the study on NSCs survival from H_2_O_2_-induced oxidative stress and its underlying mechanism have rarely been reported before. In this study, we modelled the oxidative stress injured NSCs with 100 *μ*M H_2_O_2_ induction. The oxidative induction notably increased the ROS production, detected by DCF-DA probe method. And the induction damaged the NSCs to apoptosis, even cell death, mimicking the NSCs survival in pathological environment for transplantation therapy.

Concerning the involved signalling mechanism of neural injury by oxidative stress, NF-*κ*B is normally listed as the first candidate [14, 15]. It was confirmed by our results that when induced oxidative stress happens in NSCs, NF-*κ*B is activated through IKK/I*κ*B/p65 cascaded phosphorylation. The phospho-p65 in consequence is released from the complex with I*κ*B and translocates into nucleus for relative gene transcription, which finally leads to DNA damage, signalling overactivation, and cell death. It can be considered that NF-*κ*B/p65 is involved in NSCs damage by oxidative. However, it is still too far away from concluding it as the very mechanism underlying the NSCs survival from oxidative stress.

We further move our focus to HSP90, a chaperone protein that facilities other proteins folding properly, stabilizes protein against stimulations, and rescues protein from degradation [16]. HSP90 is normally required for protein stabilization essential to tumour progress [16]. Therefore, the protein is always considered as a drug target for cancer therapy and research [17]. However, the function of HSP90 involved in neural damage and cellular oxidative stress is rarely reported wildly. Here, in this study, along with the activation of NF-*κ*B/p65 during the neural oxidative pathology, HSP90 was also triggered through upregulated protein expression and centralized distribution around nucleus. Moreover, the oxidative induced NSCs displayed an increased colocalization of HSP90 and NF-*κ*B/p65, comparing to the normal control NSCs, which suggest a cofactor relationship between the two signalling proteins on mediating oxidative stress in NSCs [18]. Previous reports described the relationship in several other pathologies as a requirement of HSP90 for IKK biosynthesis and induced NF-*κ*B activation [19] or an inhibition on NF-*κ*B/p65 by the HSP90 inhibitor [20]. To further determine the streaming relationship between the two signalling proteins, we applied their pharmacological inhibitor on each other. According to our results, the HSP90 inhibitor [21], GA, blocked the induced IKK/I*κ*B/p65 activation, while the NF-*κ*B/p65 inhibitor [12], JSH-23, failed in inhibiting HSP90 upregulation when oxidative stress happens in NSCs. It suggested an upper-stream role of HSP90 to NF-*κ*B/p65 in NSCs oxidative stress. The requirement of HSP90 for NF-*κ*B/p65 could not happen as late until p65 phosphorylation but potentially starts as early as IKK and I*κ*B activating stage, according to the effect of GA on the two upper-stream kinases of NF-*κ*B/p65. The decreased nuclear translocation of phospho-p65 and attenuated colocalization of phospho-p65 and HSP90 by GA administration followed by oxidative injury, in consistency further confirming the role of HSP90 upon NF-*κ*B/p65 activation under the pathology. As far as was determined by the relationship between HSP90 and NF-*κ*B/p65 oxidative injury, our data using GA on NSCs suggested a rescuing effect through HSP90 inhibition from oxidative stress induced cell death.

**Figure 7:**
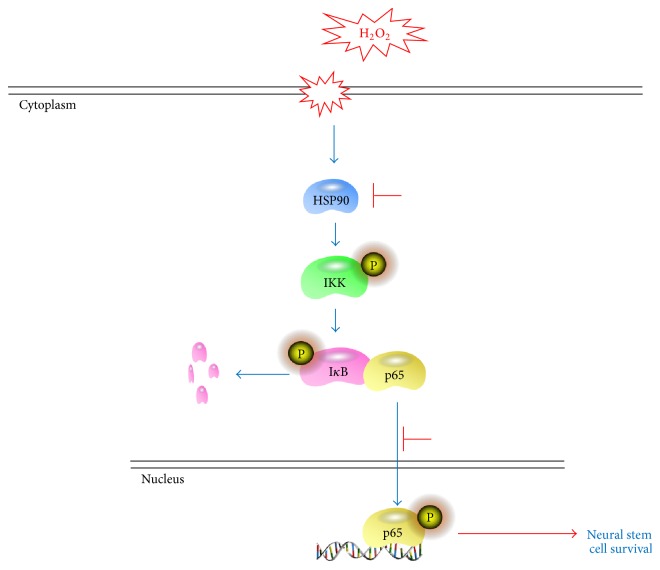
Diagram of HSP90/NF-*κ*B pathway of NSCs survival from oxidative stress. The cell death inducing oxidative stress triggers HSP90 to activate IKK/I*κ*B/p65 pathway and damage NSCs to apoptosis and death. The inhibition on HSP90 from neural oxidative induction will block the NF-*κ*B/p65 signalling activation and rescue the NSCs from oxidative damage.

In conclusion, taking together the above results, it is believed that the cell death inducing oxidative stress could trigger IKK/I*κ*B/p65 activation through HSP90 and induce NSCs damage. The inhibition on HSP90 activity from neural oxidative stress will block the NF-*κ*B/p65 signalling activation and promote the NSCs survival (Figure 7). The protecting effect on NSCs in oxidative stress and its underlying signalling mechanism including HSP90 and NF-*κ*B/p65 that we reveal in this study provides some more data on NSCs survival from oxidative stress and also promotes the stem cell study and application on CNS pathology.

## Additional Points

*Highlights.* (1) HSP90 and NF-*κ*B/p65 activation are involved in oxidative stress induced NSCs damage. (2) HSP90 plays as an upper-stream signalling of NF-*κ*B/p65 in NSCs survival from oxidative stress induced damage. (3) Inhibiting HSP90 promotes NSCs survival from oxidative stress induced damage through attenuating NF-*κ*B/p65 activation.
